# Improvement of Ficin-Based Inhibitive Enzyme Assay for Toxic Metals Using Response Surface Methodology and Its Application for Near Real-Time Monitoring of Mercury in Marine Waters

**DOI:** 10.3390/ijerph17228585

**Published:** 2020-11-19

**Authors:** Garba Uba, Motharasan Manogaran, Baskaran Gunasekaran, Mohd Izuan Effendi Halmi, Mohd Yunus Abd Shukor

**Affiliations:** 1 Department of Science Laboratory Technology, College of Science and Technology, Jigawa State Polytechnic, Dutse PMB 7040, Nigeria; garbauba@jigpoly.edu.ng; 2 Department of Biochemistry, Faculty of Biotechnology and Biomolecular Sciences, Universiti Putra Malaysia, Serdang 43400 UPM, Selangor, Malaysia; haranz715@yahoo.com; 3 Faculty of Applied Science, UCSI University, 56000 Kuala Lumpur, Selangor, Malaysia; baskaran@ucsiuniversity.edu.my; 4 Department of Land Management, Faculty of Agriculture, University Putra Malaysia, Serdang 43400 UPM Selangor, Malaysia; m_izuaneffendi@upm.edu.my

**Keywords:** inhibitive assay, mercury, ficin, RSM, near real-time

## Abstract

Potentially toxic metals pollution in the Straits of Malacca warrants the development of rapid, simple and sensitive assays. Enzyme-based assays are excellent preliminary screening tools with near real-time potential. The heavy-metal assay based on the protease ficin was optimized for mercury detection using response surface methodology. The inhibitive assay is based on ficin action on the substrate casein and residual casein is determined using the Coomassie dye-binding assay. Toxic metals strongly inhibit this hydrolysis. A central composite design (CCD) was utilized to optimize the detection of toxic metals. The results show a marked improvement for the concentration causing 50% inhibition (IC_50_) for mercury, silver and copper. Compared to one-factor-at-a-time (OFAT) optimization, RSM gave an improvement of IC_50_ (mg/L) from 0.060 (95% CI, 0.030–0.080) to 0.017 (95% CI, 0.016–0.019), from 0.098 (95% CI, 0.077–0.127) to 0.028 (95% CI, 0.022–0.037) and from 0.040 (95% CI, 0.035–0.045) to 0.023 (95% CI, 0.020–0.027), for mercury, silver and copper, respectively. A near-real time monitoring of mercury concentration in the Straits of Malacca at one location in Port Klang was carried out over a 4 h interval for a total of 24 h and validated by instrumental analysis, with the result revealing an absence of mercury pollution in the sampling site.

## 1. Introduction

The Straits of Malacca is one of the busiest waterways in the world. It has become a collection point for pollutions, including heavy metals from shipping and terrestrial activities. It is also an important contributor to marine fish production. The maximum permissible limit for Hg stipulated by the Malaysian Department of Environment under the marine water quality standards [1] is 0.040 mg/L for fisheries purposes (Class 2). The current contamination of toxic metals, including Hg in the Straits of Malacca waters, is under control. However, Wan Norhana et al. [2] has found levels of the toxic metals Cr, Zn, Cu, As and Hg in the blood cockle (*Tegillarca granosa*) or also known as *Anadara granosa* exceeding the Malaysian standards. In the West Coast of Peninsular Malaysia, Port Klang is one of the busiest ports, with its surrounding areas heavily involved with aquaculture and fishery activities, including the culture of the blood cockle (*Anadara granosa*) [3]. Being able to monitor bioavailable mercury in near real-time (less than 1 h of detection time) allows the temporal detection of toxic metals that often eludes monitoring authorities [3,4,5]. Instruments such as ICP (inductively coupled plasma) and FIMs (flow injection mercury system) are not only prohibitively expensive but are bulky for near-real time monitoring purposes. The use of rapid toxicological assays is an excellent approach for preliminary screening purposes, with only positive results sent for instrumental validation. Several rapid toxicological assays are available, including luminescence-based assays, such as Microtox^TM^ and Xenoassay light^TM^ [6,7], but they are not sensitive enough for mercury detection in marine waters at the 0.040 mg/L maximum permissible limit mentioned previously. Earlier, we have developed several toxic metals inhibition assays based on proteases, including papain [8], bromelain [9] and trypsin [10], which show promising potential in the near real-time monitoring of toxic metals [7,11,12,13,14]. The most recent development using ficin shows the most sensitive response to mercury, with an IC_50_ (concentration causing 50% inhibition) value of 0.085 mg/L [15]. However, in order to detect mercury at the limit of the MPL (0.040 mg/L) for marine waters, the assay needs to be further optimized. 

Optimization works in analytical chemistry are usually done by changing one important parameter at a time. This is officially called one-factor-at-a-time (OFAT). The most significant drawback of this approach is that the interactive portion of the factors involved is not considered. As a result, the full effects of the parameters on the response are not revealed. The increase in the number of experiments required to conduct the research is another drawback of the one-factor optimization which causes time and costs to be increased. In addition, OFAT uses more reagents and consumables [16]. Response surface methodology (RSM) is a multivariate statistical methodology capable of resolving OFAT constraints. It employs mathematical and statistical methods that can predict optimum conditions based upon the fits of experimental results to polynomial equation. RSM is particularly useful when many factors affect a set of responses of interest [17]. The goal is to maximize the rates of these variables simultaneously to produce the most optimum results. In analytical chemistry, RSM has often been used to optimize the detection of analytes in a number of cases [18,19,20]. The use of RSM in improving the sensitivity of toxic metals detection in a protease-based inhibitive assay has never been attempted, and this work, to the best of our knowledge, is the first such attempt.

## 2. Materials and Methods 

### 2.1. Preparation of Casein and Ficin Solution

Casein (Sigma) was weighed (2 g) and mixed with 100 mL of deionized water. The pH of the mixture was adjusted to 8.0 using 5 N NaOH and/or 5 N HCl. The mixture was stirred at 60 °C overnight to maximize dissolution. Several layers of cheesecloth were utilized to remove insoluble casein from the mixture. The slightly clear filtrate was further clarified by centrifugation at 10,000× *g* (4 °C). The protein content of the clear supernatant was measured using the Bradford assay with crystalline BSA (Sigma) as the standard. The solution is stored at 4 °C until further use or stored frozen at −20 °C. Ficin (SIGMA, E.C. 3.4.22.3, lot no: F4165-1ku, crude dried fig tree latex. 0.5 Units/mg) was prepared at 4 °C in 20 mM sodium phosphate pH 6.5 as a 10.0 mg/mL stock solution. Working solutions of ficin (2.0 mg/mL) and casein (10 mg/mL) were prepared from these stock solutions fresh daily.

### 2.2. Ficin Optimization Studies

Ficin activity and optimization studies based on OFAT were carried out according to previous work [15]. To match ambient temperature for field trial environment in Malaysia and to qualify for near-real-time measurement, the temperature was fixed at 30 °C and the incubation duration was fixed for 30 min [7,12,21]. 

The optimum concentration of the enzyme was studied by varying the final concentration of ficin from the stock solution to the final concentrations, ranging from 0.1 to 0.8 mg/mL in 20 mM phosphate buffer pH 6.5. Notably, 30 μL of casein was added to 50 mL of the ficin solution and the solution was mixed thoroughly. The final concentration of casein was 2 mg/mL. The volume was topped up to 150 μL using 20 mM phosphate buffer pH 6.5 and the mixture was incubated for 30 min at 30 °C. After the incubation period has elapsed, a 10 μL aliquot was immediately withdrawn and mixed with 200 μL of Bradford dye-binding reagent. After 5 min of incubation at room temperature, the absorbance at 595 nm for time zero was taken. After 30 min, another 10 μL aliquot was again taken and the absorbance at 595 nm taken (5 min incubation at room temperature) after mixing with 200 μL of Bradford dye-binding reagent. A microplate reader (Bio-Rad Model 680 microplate reader, Bio-Rad Laboratories, Inc., 3110 Regatta Blvd, Richmond, CA 94804, United States) was utilized for absorbance measurement. For optimizing the concentration of the substrate casein, ficin was fixed at 0.5 mg/mL, while casein concentrations were varied from 0.5 to 3 mg/mL in a final volume of 150 μL. To study the optimum pH for enzyme activity, ficin was set at 0.5 mg/mL and casein was set up at 2 mg/mL. A sodium phosphate buffer (20 mM) from pH 5.8 to 7.8 (±1 pKa of phosphate) was utilized, and the assay was carried out in the same manner as before, with the only difference being the pH of the assay [22]. 

### 2.3. Optimization Using RSM

#### Central Composite Design Experiments

The central composite design (CCD) was applied for the optimization of three experimental factors; namely enzyme-substrate incubation time, casein and ficin concentrations. A 2^3^ factorial central composite experimental design leading to a set of 20 experimental runs was used to optimize the detection of mercury at 0.040 mg/L. The response is the difference in the absorbance value difference of the Bradford dye-binding assay measured at 595 nm (after 20 min of incubation at 30 °C), with the greatest difference in absorbance as the most desired response. 

### 2.4. Ficin Mercury Inhibition Studies

The experiment was initiated by mixing 50 μL of ficin in 20 mM phosphate buffer pH 6.5 from the experimental runs stipulated by CCD with 50 μL of mercury (final concentration of 0.040 mg/L). The mixture was incubated for 10 min at 30 ^°^C. In the control, mercury was replaced with 20 mM phosphate buffer pH 6.5. Then, 50 μL of casein was added and mixed thoroughly (stock solution of 2.0 mg/mL). Immediately, a 20 μL aliquot was mixed with the Bradford dye-binding reagent (200 μL). The mixture was incubated at room temperature for 5 min and the absorbance was taken at 595 nm as time zero absorbance. After a 30 min incubation, another 20 μL aliquot was again taken and mixed with the Bradford dye reagent and the absorbance at 595 nm, taken after a 5-min incubation period, as before.

### 2.5. Field Trials

**Figure 1:**
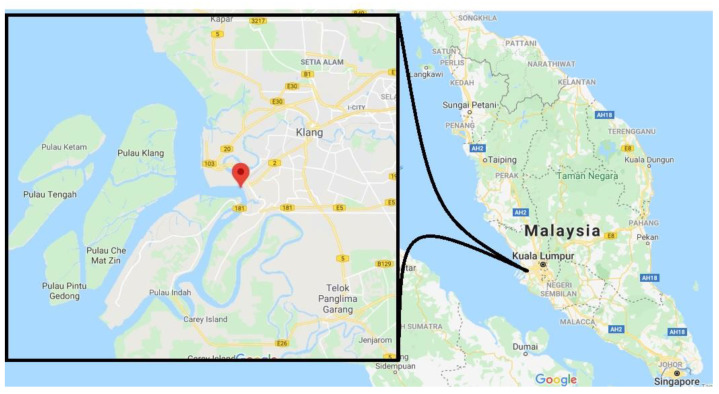
Location of marine water sampling (Source: Google Earth image).

Marine water samples were sampled periodically into acid-washed HDPE bottles containing several drops of 1% (*v*/*v*) HNO_3_ every 4 h from a location at Port Klang Selangor, with the GPS location 3°00′00.6′′ N 101°23′22.9′′ E (Figure 1). Samples were first filtered using a 0.45 µm syringe filter. Fifty microliters of the clear filtrate were immediately utilized for assaying mercury using the ficin assay at 30 °C, using a portable egg temperature incubator (30 Watt) (Generic brand name) powered by DC12 V to AC220 V Car Inverter (ZTE Avid Plus, China), capable of maintaining a temperature of 30 ± 1 °C was utilized to incubate the reaction mixture at 30 °C. The absorbance was read using a portable mini-spectrophotometer (Model M6+, Axiom, Germany). A Perkin Elmer Flow Injection Mercury System (FIMS 400) was utilized to determine the concentration of mercury, whilst silver and copper were quantified using Atomic Emission Spectrometry on a Perkin Elmer ICP OES (Optima 8300, PerkinElmer, Inc. 940 Winter Street. Waltham, MA 02451 USA). 

### 2.6. Data and Statistical Analysis

The percentage inhibition was calculated according to the following formula:

(1) $$\%\;\mathrm{Inhibition}=\frac{\mathrm{Test}\;\mathrm{activity}\;\mathrm{of}\;\mathrm{sample}-\mathrm{test}\;\mathrm{activity}\;\mathrm{of}\;\mathrm{control}}{\mathrm{Test}\;\mathrm{activity}\;\mathrm{of}\;\mathrm{control}}\times 100$$

#### Test Activity of Control

Nonlinear regression using the one-phase exponential decay model was carried out using the software GraphPad Prism (Trial version 8.0.2). Means and standard deviations were determined according to at least three independent experimental replicates. RSM was carried out using the trial version of the Design-Expert version 7.0 software (Stat-ease Inc., USA).

## 3. Result and Discussion

### 3.1. Optimization Using OFAT

**Figure 2:**
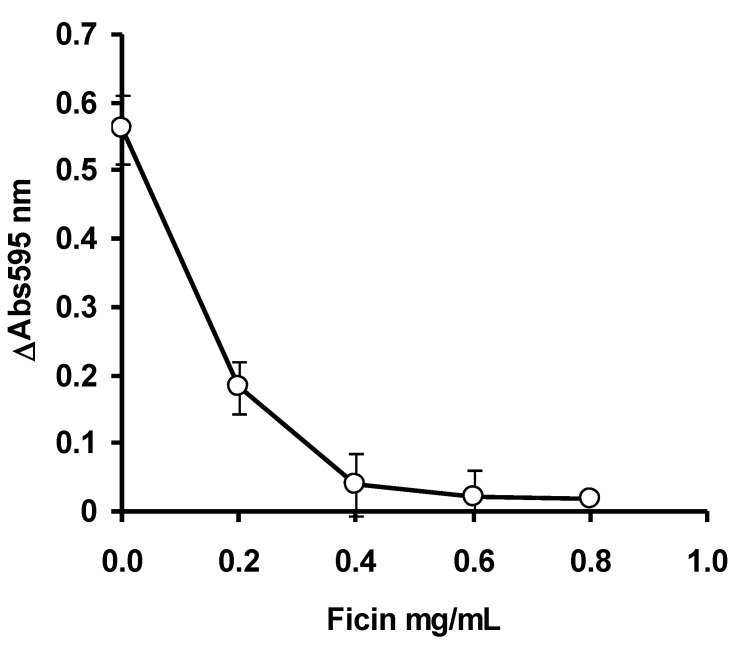
The effect of ficin concentration on the ficin dye-binding assay. Error bars represent mean ± standard deviation (*n* = 3).

**Figure 3:**
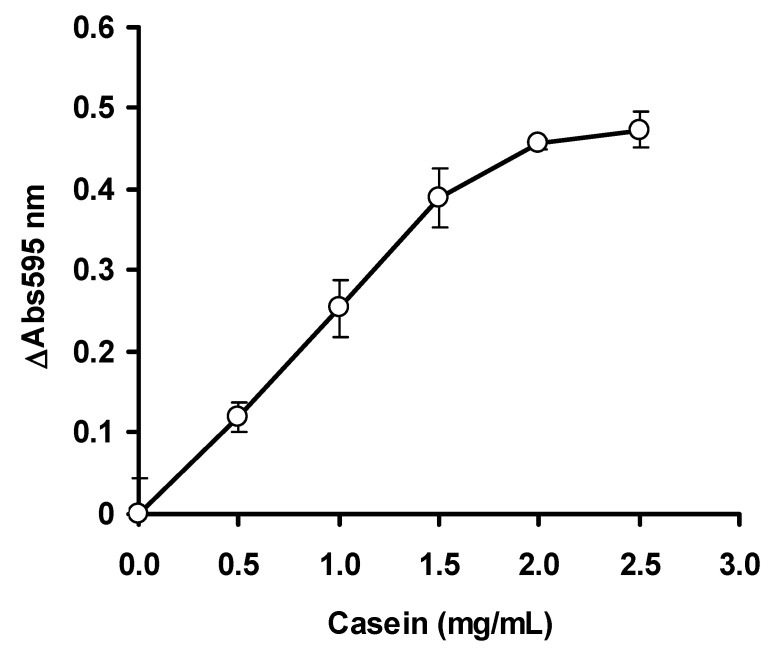
The effect of casein concentration on the ficin dye-binding assay. Error bars represent mean ± standard deviation (*n* = 3).

**Figure 4:**
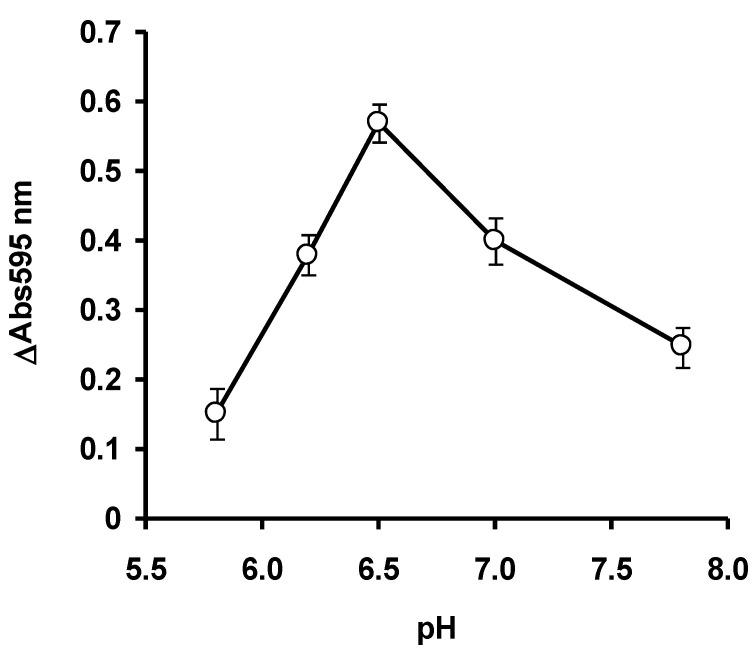
The effect of pH on the ficin dye-binding assay. Error bars represent mean ± standard deviation (*n* = 3).

The ficin assay mercury is an inhibitive assay. Under control (no mercury) situation, the ficin will degrade the substrate casein leaving oligopeptides (<2 kDa) that the Bradford assay cannot react, and the solution remains brown. In the presence of mercury, ficin is inhibited and the unreacted casein will be detected by the Bradford assay, leading to an intense blue solution. The optimization studies via OFAT showed that ficin activity was optimum at 0.4 mg/mL ficin (Figure 2), 2.0 mg/mL of casein (Figure 3) and at pH 6.5 (Figure 4). In the papain inhibitive assay for mercury, OFAT optimization gave the best combination of enzyme and casein, both at 0.1 mg/mL [8]. In the bromelain assay, 0.11 mg/mL bromelain and 0.25 mg/mL casein are the best combinations [9]. Generally, protease assays including ficin can utilize a variety of substrates, including natural such as casein and azocasein, and artificial substrates such as Nα-benzoyl-L-arginine-p-nitroanilide (BAPNA). Similarly, other protein assays such as Biuret–Lowry, Folin–Ciocalteu and bichinconic acid can replace the Bradford assay. Despite this, the Bradford dye-binding assay continues to be a popular assay, for a number of reasons, including rapidity, sensitivity simplicity, and most importantly, it is the most robust, and can be used in the presence of many interfering compounds [23]. The mechanism behind the inhibition studies of this ficin enzyme is very much related to its active site. Since this enzyme is a cysteine protease, it has SH group in its active site. Without the presence of inhibitive toxic metals, the enzyme can function normally in the degradation of the substrate casein and thus it gives a light-brownish color when stained with Bradford. The presence of silver, mercury and copper which bind to the SH group of the active site inhibits the hydrolysis of casein. The binding of toxic metals also causes conformational changes of the active site and substrates can no longer be fitted. The unhydrolyzed casein is then stained by the Bradford dye-binding reagent to dark blue [8,9].

### 3.2. Optimization Using Response Surface Methodology (RSM)

The central composite design (CCD) was employed to study the optimum concentration of the factors (Table 1). Twenty experiments were designed by the Design expert software 6.0 with six replicates of midpoints, which are useful to determine the experimental error (Table 2).

The regression equation and the determination coefficient (*R^2^*) were assessed to test for the model fitness (Table 3). The model F-value of 899.05 is an indication that the model was significant. There is only a 0.01% chance that a “model F-value” this large might happen as a result of noise alone. It was observed that the model had a low probability value (<0.0001) and a lack of the fit test F value of 1.435 (non-significant), implying that the model was fit. Insignificant lack of fit is important, since it means that the observational errors are not significant, directed and systematic [24,25,26]. The "Pred R-Squared" of 0.9929 is within reasonable agreement with the "Adj R-Squared" of 0.9977.

The reliability of the experiments can be further established with a low coefficient of variance (CV) value [27]. The CV of 4.3% for this study is an indication of the reliability of experiments performed. The significance of regression for the coefficients was considered. In this case, A, B, C, A^2^, B^2^, C^2^, AB, AC and BC were the significant model terms (Table 3). Hence, a statistical analysis of the experimental data revealed that all three factors had a significant effect throughout the study. 

**Figure 5:**
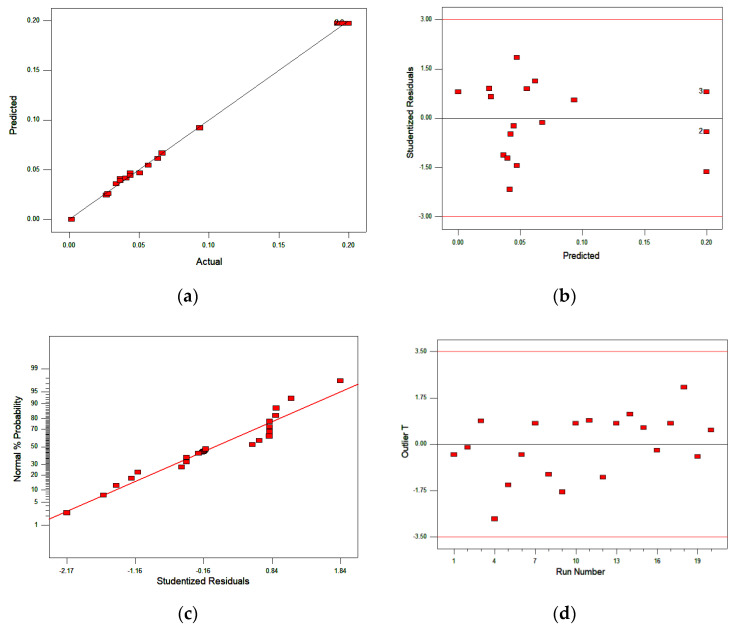
Model diagnostic plots; (**a**) predicted versus actual, (**b**) studentized residue versus predicted, (**c**) normal plots of residue and (**d**) outlier T versus run.

The fitness of data into the selected model was examined through diagnostic model plots (Figure 5a–d). The plots are important, particularly in the assessment of data error that differs from model predictions, which aids in assessing and improving model adequacy [27]. The plot of actual versus predicted values obtained from the experiment (Figure 5a) revealed a close relationship between the actual and predicted value, as the data points assembled close to the line that divides the plot into equal halves (45°). The adequacy of the model was further verified by plotting the predicted values and studentized residuals (Figure 5b). Studentized residues are a variation between the predicted value and actual responses obtained from the model. The plot of normal probability demonstrates slight or no abnormality in the experimental data (Figure 5c). An outlier plot (Figure 5d) visualizes the distantly standout standard deviation of actual response from the rest of the data. No outlier was evident from the plot, as all the data fall between 3.5 and −3.5.

**Figure 6:**
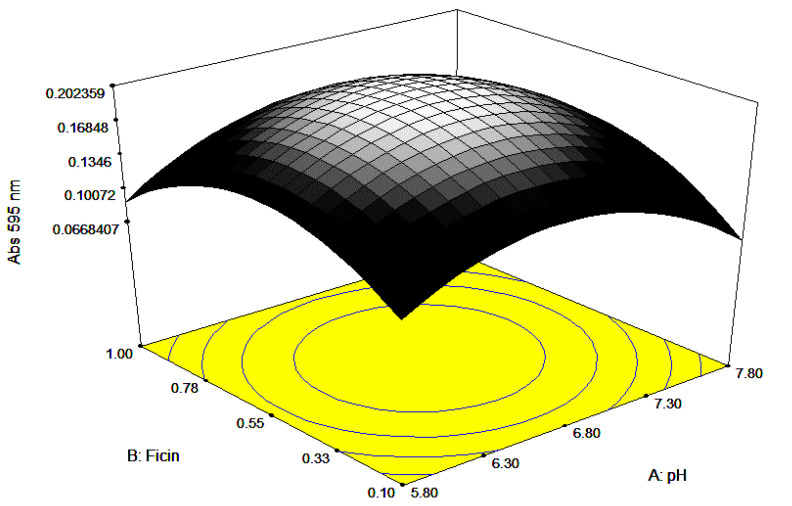
Here, 3D surface response view showing the response when ficin concentration and pH were varied.

**Figure 7:**
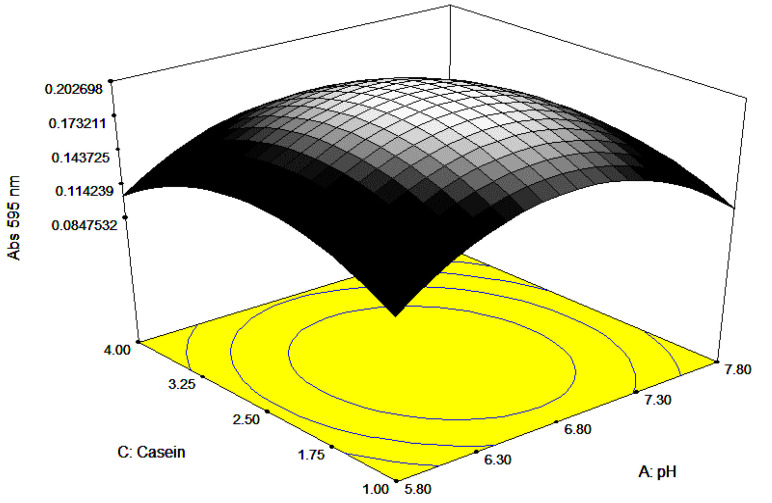
Here, 3D surface response view showing the response when casein concentration and pH were varied.

**Figure 8:**
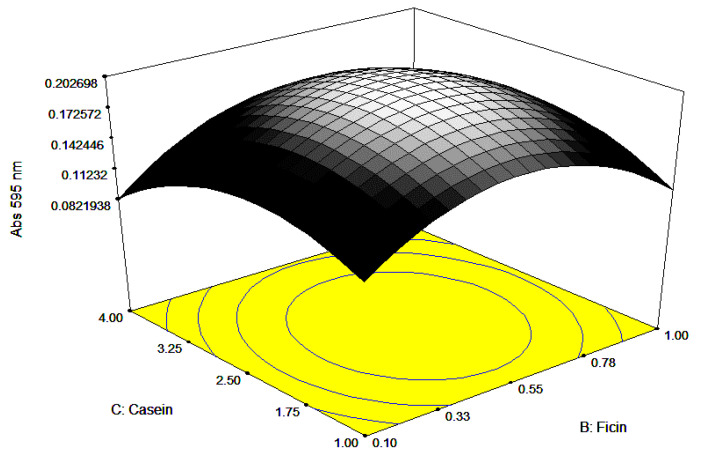
Here, 3D surface response view showing the response when ficin and casein concentrations were varied.

Visualization of all the factors required for maximum growth is presented through 3-dimensional responses and contour plots (Figure 6, Figure 7 and Figure 8). The plots are of the utmost importance in determining the relationship at zero or intermediate levels of different combinations of independent factors before performing a real experiment [28,29]. The 3D response plots show the maximum response between each pair of a factor, while the other factor is held constant. The curved contour lines imply interaction, with elliptical or saddle contour plots represent significant interaction, whilst circular contour plots suggest that interaction is not significant. Mathematically, interaction can be uncovered looking at the numerical value in the coded equation, where a large numerical value, either negative or positive values, designates that significant interaction occurred [30]. The perturbation plots, 2D plot for the combination of factors, show the interaction between the factors (results not shown), especially between ficin and pH and casein and ficin concentrations. Normally, the interaction between two different factors occurs when there is a different response obtained, when varying the outcome of one factor at different levels compared to the other factor [31]. As the perturbation lines amongst the ficin concentration and pH, and ficin concentration and casein concentration did not cross each other, this suggests that the interaction is synergistic instead of antagonistic [30].

An improvement of absorbance value (A595 nm) of 0.212 ± 0.04 was obtained after RSM exercise, compared to OFAT, which gave an absorbance value of 0.045 ± 0.014, suggesting that the use of RSM was successful. The model equation fitted by regression analysis is given (coded factor) as follows:

Abs 595 nm = 0.20 − 5.092E − 003 * A + 5.328E − 003 * B − 7.575E − 003 * C − 0.058 * A * A − 0.057 * B * B − 0.042 * C * C + 9.750E − 003 * A * B − 4.750E − 003 * A * C + 8.250E − 003 * B * C(2)

Actual factor (inverse relationship with coded factor);

Abs 595 nm = −2.60870 + 0.78328 * pH + 0.14405 * Ficin + 0.10293 * Casein − 0.058263 * pH^2^ − 0.28191 * Ficin * Ficin − 0.018634 * Casein * Casein + 0.021667 * pH * Ficin − 0.00032 * pH * Casein + 0.012222 * Ficin * Casein(3)

### 3.3. Comparison of OFAT and RSM in Mercury Detection Using Ficin 

**Figure 9:**
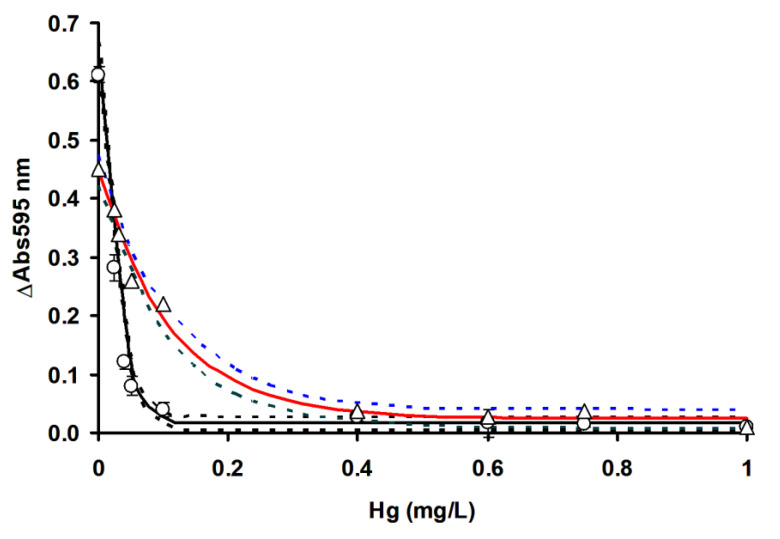
Ficin dye-binding assay of mercury using RSM (◯) and OFAT (△). Dashed lines represent the 95% confidence band. Error bars represent mean ± standard deviation (*n* = 3).

**Figure 10:**
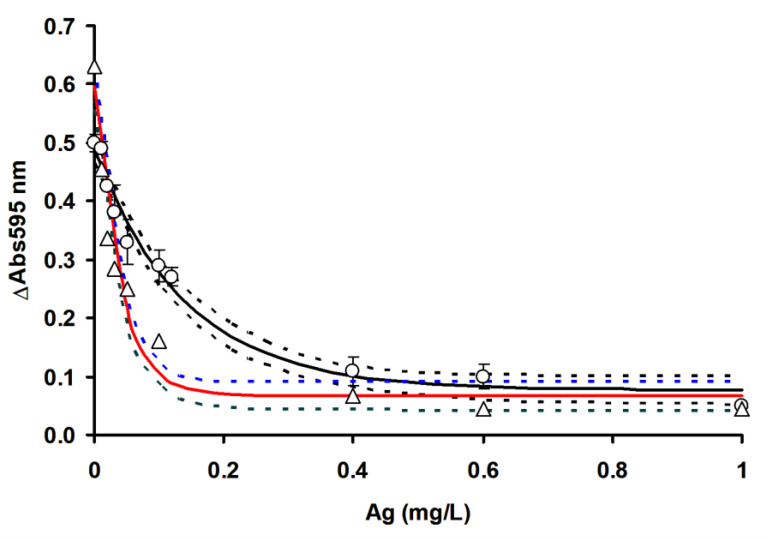
Ficin dye-binding assay of silver using RSM (◯) and OFAT (△). Dashed lines represent the 95% confidence band. Error bars represent mean ± standard deviation (*n* = 3).

**Figure 11:**
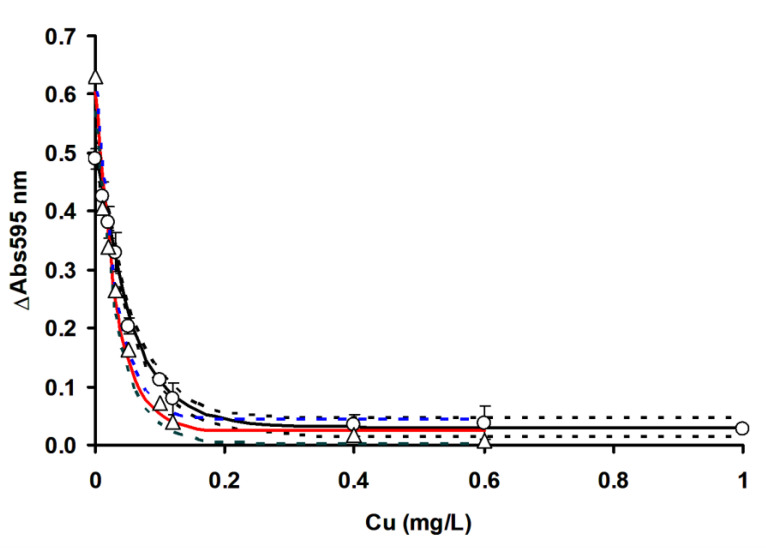
Ficin dye-binding assay of copper using RSM (◯) and OFAT (△). Dashed lines represent the 95% confidence band. Error bars represent mean ± standard deviation (*n* = 3).

In order to compare the efficacy of RSM in optimizing the detection of mercury, the ficin Coomassie dye-binding assay for mercury (Figure 9), silver (Figure 10) and copper (Figure 11) is compared to OFAT by constructing the inhibition curve and the results of the IC_50_ values are shown in Table 4. 

IC_50_ value refers to the concentration of toxic metals that inhibits the enzyme activity by 50%, while LOD is the limits of detection, which is three times the standard deviation of the blank. The non-overlapping confidence interval of parameter estimates in nonlinear regression generally indicates a significant difference at the alpha value utilized (0.05), whilst an overlapping CI does not, in general, indicate significance or non-significance; more data is needed to reach a conclusion. In this work, we only consider non-overlapping CI as a benchmark for indicating process optimization (RSM) success in improving the sensitivity of toxic metals’ detection using the ficin assay, and in this case, RSM managed to improve the LOD and IC_50_ values for all the toxic metals tested. Notably, the use of RSM increased the A595 nm values by 0.1 absorbance value on average, which is a marked improvement. The use of RSM in analytical works to improve the sensitivity of detection has been documented in many cases [18,19,20,32,33,34], and the improving of the sensitivity of toxic metals in the ficin assay is a testament to the utility of RSM. In this study, we utilized the one-phase exponential decay model, which gave good fitting to the experimental data, with the coefficient of determination (*R^2^*) values ranging from 0.9545 to 0.995.

Whether a developed assay is more sensitive to other existing assays can be assessed based on the overlapping or non-overlapping of the confidence interval of the IC_50_, as discussed previously. The ficin IC_50_ for copper was much lower than 15-min Microtox, 96-h Rainbow trout, bromelain, acetylcholinesterase (AChE) from *Pangasius* sp. and the Mo-reducing enzyme from *Serratia* sp. strain DrY8 assays. It is similar in sensitivity to the 48-h *Daphnia magna* and less sensitive to the papain assay. For mercury, the ficin assay is more sensitive than all other assays, with the exception of 48-h *Daphnia magna*, where it is comparable in sensitivity. For silver the ficin assay is more sensitive than all other assays, with the exception of the Mo-reducing enzyme from *Serratia* sp. strain DrY8, where it is comparable in sensitivity (Table 5).

### 3.4. Near Real-Time Field Trials

**Figure 12:**
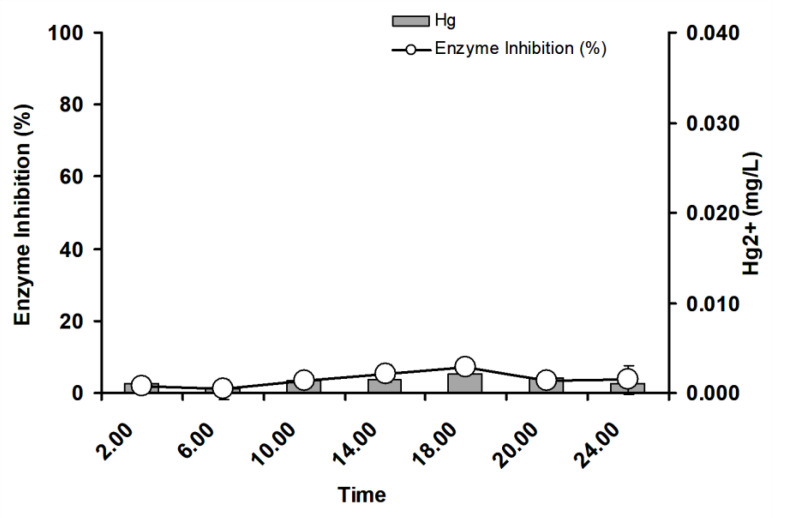
Near real-time detection of mercury in Port Klang waters using the ficin inhibitive enzyme assay. Presence of mercury was validated using FIMS. Error bars represent mean ± standard deviation (*n* = 3).

A near real-time field trial was carried out in Port Klang, in the state of Selangor. The area is characterized by significant and rapid economic growth, including harbors, ports, heavy industries, commercial sites, residential, tourism and fishery activities. There is negligible inhibition (<10%) to the ficin assays, and an instrumental analysis shows mercury level lower than the designated MPL in marine waters (0.040 mg/L) (Figure 12). Other near real-time works using enzymes in rivers show temporal levels of toxic metals [7,11,14,15,38], and this is the first study using marine water as samples. Marine waters are large bodies of water where toxic metals originating from terrestrial areas are rapidly diluted. Elevated levels of toxic metals have been found in this region, but most are found in the sedimental fractions [39]. This could explain the lack of inhibition to the ficin and a lower concentration of mercury in marine waters within this area.

Variation in toxic metals levels in running water, especially in rivers and marine water bodies, is common [7,11,12,13,14,40]. Even sedimentary samples have been found to be variable in their spatial and temporal concentrations of toxic metals [41]. This variability requires a fast detection system to capture the temporal variation in toxic metals’ concentrations, as this is important in environmental forensic applications. The current detection system can best be described as a batch system, with samples needed to be transported to the laboratory prior to the determination of their toxic metals content [8,36,42]. One of the solutions to this problem is the real-time or near real-time monitoring of toxic metals. The use of bioassays involving plants, microorganisms and enzyme assays can address this issue [43,44,45]. In enzyme assays, the sampling to detection period can be carried out in less than one hour using a portable spectrophotometer, and thus is a perfect candidate for near real-time analysis. We have demonstrated the application of enzyme-based system in capturing in near real-time the temporal variation of toxic metals concentrations in rivers running through heavily industrialized areas [7,11,12,13,14]. The application of the ficin assay in monitoring mercury in marine water bodies in this study is a novel exercise meant as a proof of concept. More sampling locations need to be identified and more field trials will be carried out in the future.

## 4. Conclusions

The use of RSM based on CCD was successful in optimizing the protease ficin dye-binding assay for mercury, silver and copper, resulting in a more sensitive determination of these metal ions. The resultant LOD and IC_50_ values were better than the values obtained with the OFAT approach. The sensitivity of mercury, in particular, was good enough to detect mercury at the maximum permissible limit allowed for marine waters. The developed assay was then tested as a near real-time assay for the detection of mercury from a marine site in Port Klang. Mercury was not detected in this site at all of the sampling periods, indicating the absence of pollution due to mercury in this water. More samples from diverse sampling points are currently being tested to monitor the presence of toxic metals, especially mercury in coastal areas and rivers in Malaysia. The assay is rapid, sensitive, easy to be carried out and has the ability to monitor in near real-time as a preliminary screening tool for toxic metals pollution.

## Figures and Tables

**Table 1:** Coded and actual values of significant factors used in central composite design (CCD).

Figure	Name	Units	Type	Low Actual	High Actual	Low Coded	High Coded
A	pH	min	Numeric	5.8	7.8	−1	1
B	Ficin	mg/mL	Numeric	0.1	1	−1	1
C	Casein	mg/mL	Numeric	1	4	−1	1

**Table 2:** CCD experimental matrix generated by the Design expert software and the corresponding responses (actual and predicted).

Run.	pH	Ficin (mg/mL)	Casein (mg/mL)	Response (Abs595 nm)	Predicted (Abs595 nm)
1	6.8	0.55	2.5	0.201	0.200
2	6.8	0.55	5.02	0.071	0.071
3	7.8	1	1	0.061	0.059
4	7.8	0.1	1	0.041	0.045
5	7.8	1	4	0.048	0.051
6	6.8	0.55	2.5	0.201	0.200
7	6.8	0.55	2.5	0.205	0.200
8	5.8	1	1	0.038	0.040
9	6.8	0.55	2.5	0.197	0.200
10	6.8	0.55	2.5	0.205	0.200
11	8.48	0.55	2.5	0.031	0.029
12	5.8	0.1	4	0.041	0.044
13	6.8	0.55	2.5	0.205	0.200
14	5.8	0.1	1	0.068	0.066
15	6.8	0.21	2.5	0.032	0.031
16	6.8	1.31	2.5	0.048	0.049
17	7.8	0.1	4	0.006	0.004
18	5.8	1	4	0.055	0.051
19	5.12	0.55	2.5	0.045	0.046
20	6.8	0.55	−0.02	0.098	0.097

**Table 3:** Analysis of variance (ANOVA) for the Response Surface Quadratic Model.

	Sum of		Mean	F		
Source	Squares	DF	Square	Value	Prob > F	
Model	0.105101	9	0.011678	899.0521	<0.0001	significant
A	0.000354	1	0.000354	27.26442	0.0004	
B	0.000389	1	0.000389	29.95455	0.0003	
C	0.000783	1	0.000783	60.27813	<0.0001	
A^2^	0.048926	1	0.048926	3766.679	<0.0001	
B^2^	0.047597	1	0.047597	3664.359	<0.0001	
C^2^	0.025238	1	0.025238	1943.044	<0.0001	
AB	0.000761	1	0.000761	58.54923	<0.0001	
AC	0.000181	1	0.000181	13.8963	0.0039	
BC	0.000544	1	0.000544	41.91986	<0.0001	
Residual	0.00013	10	1.3 × 10^−5^			
Lack of Fit	7.66 × 10^−5^	5	1.53 × 10^−5^	1.435451	0.3506	not significant
Pure Error	5.33 × 10^−5^	5	1.07 × 10^−5^			
Cor Total	0.105231	19				

**Table 4:** Comparison of Limits of Detection (LOD) and IC_50_ values for toxic metals assayed using the ficin dye-binding assay obtained using RSM and OFAT. Values in parentheses indicate 95% confidence interval.

	OFAT		RSM	
Metals	LOD (mg/L)	IC_50_ (mg/L)	LOD (mg/L)	IC_50_ (mg/L)
Hg^2+^	0.018 (0.013 to 0.023)	0.060 (0.030 to 0.080)	0.002 (0.001 to 0.004)	0.017 (0.016 to 0.019)
Ag^+^	0.010 (0.007 to 0.018)	0.098 (0.077 to 0.127)	0.003 (0.002 to 0.004)	0.028 (0.022 to 0.037)
Cu^2+^	0.019 (0.016 to 0.021)	0.040 (0.035 to 0.045)	0.002 (0.001 to 0.004)	0.023 (0.020 to 0.027)

**Table 5:** Comparison of the developed ficin dye-binding assay to various other assays. The range is 95% Confidence Interval.

LC_50_ or IC_50_ (mg/L)								
Metals	15-min. Microtox^TM a, c^	48 h *Daphnia magna* ^a^	96 h Rainbow trout ^a c^	Papain ^b^	Bromelain ^d^	AChE from *Pangasius* sp. ^d^	This Study	Mo-Reducing Enzyme from *Serratia* sp. Strain DrY8 ^e^
Cu^2+^ Hg^2+^ Ag^+^	0.076–3.8 0.029–0.050 n.i.	0.020–0.093 0.005–0.21 1.930	0.25 0.033–0.210 0.050	0.004 0.24–0.62 0.33–0.49	0.163–0.305 0.13–0.16 n.i.	0.065–0.096 0.059–0.088 0.082–0.095	0.020–0.027 0.016–0.019 0.022–0.037	0.295–0.435 0.154–0.178 0.018–0.046

Note ^a^ ref [35]; ^b^ ref [8]; ^c^ ref [36]; ^d^ ref [9]; ^e^ ref [37]; n.i no inhibition.
